# Novel statistical approach for assessing the persistence of the circadian rhythms of social activity from telephone call detail records in older adults

**DOI:** 10.1038/s41598-020-77795-4

**Published:** 2020-12-08

**Authors:** Timothée Aubourg, Jacques Demongeot, Nicolas Vuillerme

**Affiliations:** 1 Univ. Grenoble Alpes, AGEIS, Grenoble, France; 2 Orange Labs, Meylan, France; 3 LabCom Telecom4Health, Univ. Grenoble Alpes & Orange Labs, Grenoble, France; 4 Institut Universitaire de France, Paris, France

**Keywords:** Computational science, Statistics, Human behaviour

## Abstract

How circadian rhythms of activity manifest themselves in social life of humans remains one of the most intriguing questions in chronobiology and a major issue for personalized medicine. Over the past years, substantial advances have been made in understanding the personal nature and the robustness—i.e. the *persistence*—of the circadian rhythms of social activity by the analysis of phone use. At this stage however, the consistency of such advances as their statistical validity remains unclear. The present paper has been specifically designed to address this issue. To this end, we propose a novel statistical procedure for the measurement of the circadian rhythms of social activity which is particularly well-suited for the existing framework of persistence analysis. Furthermore, we illustrate how this procedure works concretely by assessing the persistence of the circadian rhythms of telephone call activity from a 12-month call detail records (CDRs) dataset of adults over than 65 years. The results show the ability of our approach for assessing persistence with a statistical significance. In the field of CDRs analysis, this novel statistical approach can be used for completing the existing methods used to analyze the persistence of the circadian rhythms of a social nature. More importantly, it provides an opportunity to open up the analysis of CDRs for various domains of application in personalized medicine requiring access to statistical significance such as health care monitoring.

## Introduction

Circadian rhythms are endogenous processes characterized by a period close to 24 h depending on individuals^1^. Their ubiquity makes them one of the most perceptible phenomena in an individual’s life, reflecting and affecting all his fundamental domains of activity: biological, physical and social^2–4^. In the field of health care monitoring, the analysis of the biological, physical and social mechanisms involved in the emergence, maintaining and characterization of the circadian rhythms does represent an increasingly important issue. In medicine, this importance was evidenced by the recent awarding of Nobel Prize for Medicine in 2017 to Michael Young, Michael Rosbash and Jeffrey Hall for their discoveries into the molecular mechanisms controlling the circadian rhythms^5^. From a clinical perspective, it is now well recognized that the deep comprehension of the circadian rhythms represents an opportunity for better managing a patient’s health in time^6^. In particular, for the clinical practice, this comprehension can help the health professional properly address his patients’ needs and care by delivering the adequate treatment at the optimal time of day^7^. For several decades now, such an interest for the analysis of the circadian rhythms of activity has been present in the ever-growing literature on subjects related to their biological and physical manifestations and on their association with health outcomes (see^8–10^ for recent reviews).

Nowadays, the digital ubiquity characterizing our ‘hyper-connected’ society brings new paradigms for addressing the understanding of circadian rhythms in health. In particular, while the study of the biological and physical mechanisms controlling the circadian rhythms is well addressed yet by, respectively, the fields of chronobiology and that of actigraphy, a new ubiquitous computing paradigm has emerged for addressing their social manifestations. This paradigm sets on the observation that modern technologies, and phone technologies particularly, are now completely disseminated inside our daily social lives^11^. Accordingly, the analysis of their generated data could help to better model and understand the social aspects of an individual’s behavior at a daily scale^11^. From a clinical perspective, this approach is of strong interest given that the social manifestations of circadian rhythms are, evidently, not easily perceptible for the biological and physical approaches currently used in chronobiology and actigraphy.

Along these lines, a recent body of literature has emerged around the use of phone technologies for social and behavioral modeling^12–23^. On the whole, this literature emphasizes the relevance of call detail records (CDRs)—which synthetize telephone calls and SMS exchanges of a telephone user—for the analysis of the circadian rhythms of social interactions that occur at telephone^15^. In particular, it is evinced that CDRs analysis permit to investigate continuously, objectively and unobtrusively, essential properties of such social rhythms^24–28^. Following this train of thought, recent works have reported how the persistence in time of social interactions occurring during the day at telephone could possibly be one of these properties^24–27^. For a given individual, persistence is considered as the maintaining in time of the robustness and distinctiveness of a measured phenomenon at telephone, also named *pattern*, against a comparative population^21^. A measured phenomenon that is characterized as persistent for an individual is then considered as a *signature* of his telephone activity^21^.

The first persistence analysis applied to telephone call activity was carried out by *Saramäki *et al*.* from the Aalto University in Finland in 2014^21^. In their PNAS article, these authors reported on an 18-month CDRs dataset of 30 students the existence of social signatures in the way students allocate their volume of communications with their social network over successive months. In short, each student was found to present a pattern of communicating with his social network which varies from one to another. And this pattern was found to be maintained in time despite the occurrence of a major social turn-over induced by high-school-to-university or high-school-to-work transitions. Following the methodology described by *Saramäki *et al*.*, *Alessandretti *et al*.* reported on 850 high resolution trajectories and call detail records of participants in a 24 months longitudinal experiment, the Copenhagen Networks Study (CNS)^29^, the existence of social and spatial signatures over months^30^. In particular, they show how CDRs analysis permits to evidence persistence in the way an individual exploits known assets in the social and spatial spheres. Finally, for the circadian rhythms, *Aledavood *et al*.* reported on the same dataset as that used in^21^ the existence of temporal signatures in the way participants distribute their frequency of outgoing calls according to the hour of day^25^. Other studies further show how these persistent circadian patterns can be also evidenced with other types of social interactions^26,27^, such as text messages^26^, or with other populations as the older one^24,28^.

In the field of health care monitoring, such results on the robustness and distinctiveness of the digital signatures of telephone activity can permit to better understand the social elements involved in the circadian rhythms of activity that are of a social and personal nature. These elements can be used for improving health care monitoring by informing health professionals on the structure and quality of a patient’s daily rhythm of his social activity at telephone. In the research community, such an interest in the analysis of the daily rhythms of telephone activity is well highlighted by recent studies on this topic^24,28,31–34^ and has contributed to the emergence and success of new promising innovative fields in health, as reflected by the digital phenotyping one^35^, the field of digital psychiatry^36^ or that of mobile health (*mHealth*)^13^ just for naming the most cited ones (see the work shared in the Lancet journal by a recent commission on the future of psychiatry for instance^36^).

At this stage however, concerning the persistent nature of the digital activity measured from phone technologies, it must be said that the statistical validity of these promising results remains unclear. In^21^ for instance, the analytical methodology used for assessing the robustness and distinctiveness of individual patterns of telephone call activity involves mean estimators only and no statistical validation. Similar estimators were used in^30^, in conjunction with the following decision rule: given a phenomenon observed at telephone for an individual $i$ from a population $A = \{ j , j \in \left[ {1 \ldots ,i, \ldots ,M} \right])$, if its distinctiveness over time is validated “for most j [with $j \ne i$], we can conclude that for individual $i$, fluctuations of the [measured phenomenon] are negligible compared to the difference with other individuals”. This method is exactly the same as that used for assessing the persistent nature of the circadian rhythms of telephone call activity, which is reported in^24–27^.

For the clinical practice, assessing the statistical significance of the persistent nature of the circadian rhythms of telephone call activity is essential before validating their consistency. Thus, at this stage, whether and how the existing results reported in the literature on the subject could be validated statistically and within an appropriate methodology remains to be established. The present paper has been specifically designed to address this issue. To this end, we introduce a novel statistical approach for measuring the persistence of the circadian rhythms of activity at telephone which is particularly well-suited for the existing framework of persistence analysis^21^. Then, we illustrate how this procedure works concretely by assessing the persistence of the circadian rhythms of telephone call activity on a 12-month CDRs dataset of adults over than 65 years. Three cases are tested separately: (1) outgoing, (2) incoming, and (3) total call activities. The results are discussed and, at the end, a future direction is proposed.

## Methods

### Study population and data collection

This study is based on 12 months of CDRs for 26 volunteers (20 women, 6 men; median age: 84 years; range: 71–91 years). CDRs provided by the local communication service provider were collected from the personal telephone(s) of the volunteers. Each CDRs contains the date, hour, source ID, recipient ID, direction, and duration of call (in seconds). Note that the telephone owners and the telephone contacts remained anonymous. The present study and its corresponding experimental protocols were declared to the French Data Protection Authority (CNIL registered data protection officer, France Telecom 2011 n°44). All experimental methods were carried out as per the relevant regulations and written informed consent was obtained from all participants before data were collected and anonymized. This study is secondary analysis of previous publised and unpublished data studies^28,37^.

### Data analysis

**Figure 1:**
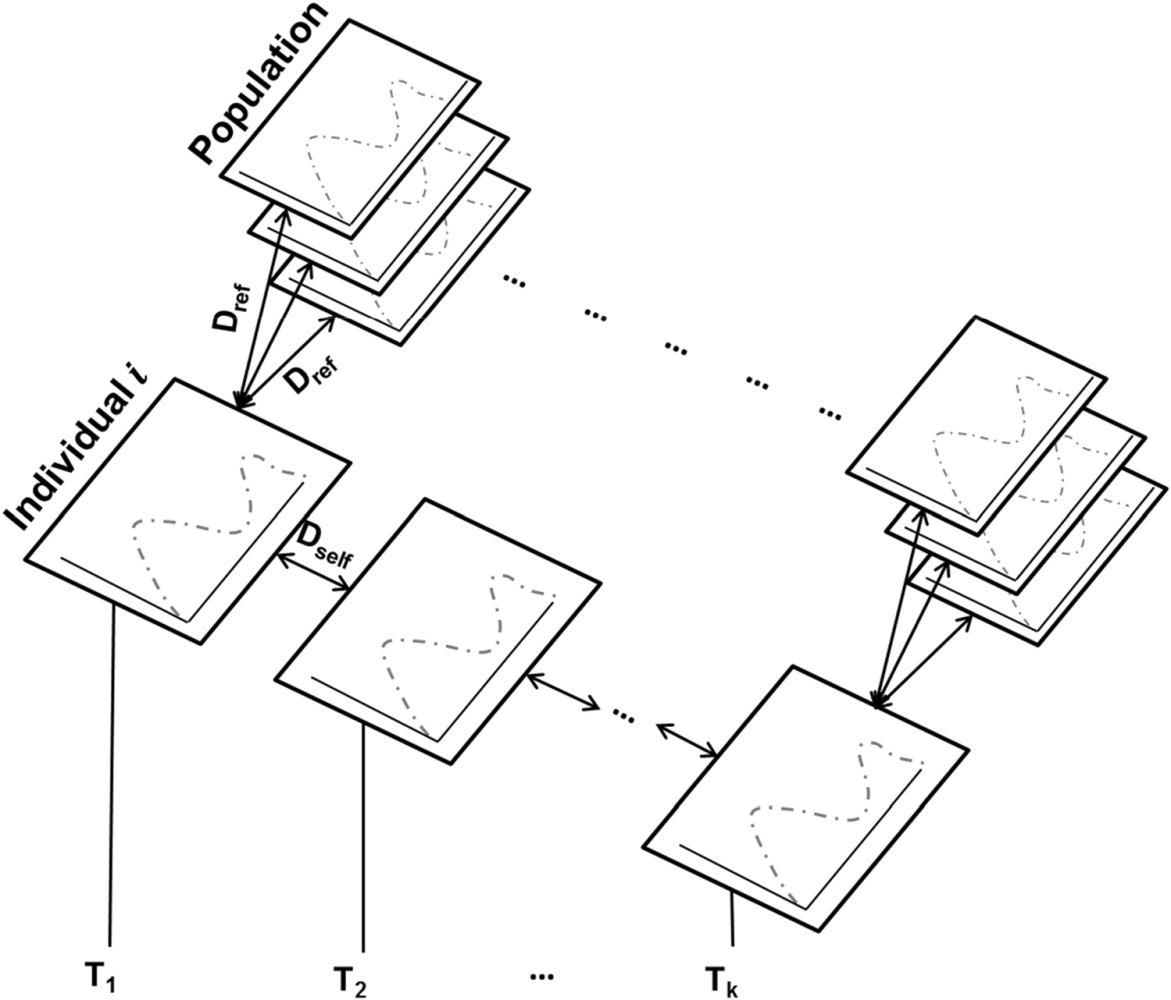
Illustrative view of the persistence analysis process applied to the circadian rhythms of activity. Here, D_self_ corresponds to the dissimilarity measured between the daily rhythms of two successive periods of time (which vary from T_1_ to T_k_) of an individual $i$, named *intra-individual* dissimilarity. D_ref_ correspond to the dissimilarities measured between the daily rhythms of an individual *i* and the other individuals of the observed population within a same time period, named *inter-individual* dissimilarities. Persistence is validated if and only if D_self_ that is measured between two successive time windows tends to minimize D_ref_’s that are measured for each of these two time windows separately.

The statistical procedure used for assessing the persistence of the circadian rhythms of telephone call activity relies on a method originally proposed by *Saramäki *et al*.* in their PNAS article^21^, and which is illustrated on Fig. 1.

Here, we propose both to formalize this existing method into a broader analytical procedure that introduces supplementary steps for ensuring the statistical validation of results (step 5.1, step 5.2 and step 5.3). Furthermore, this procedure distinguishes two cases of analysis: (1) one that compares two successive temporal windows, and (2) another that extends case (1) for comparing more than two successive temporal windows.

### Statistical procedure

#### Persistence analysis for two successive time periods (N_T_ = 2)

Let a given population of $n$ individuals $A = \left\{ {i, i \in \left[ {1, \ldots ,n} \right]} \right\}$ observed over two successive periods of time T_1_ and T_2_. The persistence analysis consists of the five following steps:

**Step 1:** *Time discretization *The individuals’ CDRs are coarse-grained into two successive periods of time, T_1_ and T_2_. Each of these two periods is divided into 24 one-hour time slots.

**Step 2:** *Calculation of daily rhythm* For each individual $i$ from population $A$, the daily rhythm of telephone calls is calculated for each period T_1_ and T_2_ by using the function $f_{i} \left( t \right) = \frac{{n_{i} \left( t \right)}}{{\mathop \sum \nolimits_{t = 0}^{23} n_{i} \left( t \right)}}$, where $n_{i} \left( t \right)$ is the number of calls in time slot $t$ of individual $i$, with $t \in \left[ {0 \ldots 23} \right]$.

**Step 3:** *Intra-individual dissimilarity *We denote by $D_{{{\text{self}}}}$ the dissimilarity measure of the individual’s daily rhythms between T_1_ and T_2_. $D_{{{\text{self}}}}$ is given by:

$$D_{{{\text{self}}}} \left( {i,{\text{T}}_{1} ,T_{2} } \right) = \sqrt {{\text{D}}\left( {i,P_{i}^{{{\text{T}}_{1} }} ,P_{i}^{{{\text{T}}_{2} }} { }} \right)} ,$$

where ${\text{D}}$ is a dissimilarity measure,$P_{i}^{{{\text{T}}_{1} }}$ ($P_{i}^{{{\text{T}}_{2} }} )$ is the discrete probability distribution of the call fractions for individual *i* calculated at time period T_1_ (T_2_), in step 2.

Finally, we denote $y_{i} = D_{{{\text{self}}}} \left( {i,{\text{T}}_{1} ,{\text{T}}_{2} } \right)$.

**Step 4:** *Inter-individual dissimilarity.* We denote by $D_{{{\text{ref}}}}$ a dissimilarity measure between two daily rhythms for two distinct individuals in the same time period. $D_{{{\text{ref}}}}$ is given by:

$$D_{{{\text{ref}}}} \left( {i,j,{\text{T}}_{{\text{k}}} } \right) = \sqrt {{\text{D}}\left( {P_{i}^{{{\text{T}}_{{\text{k}}} }} ,P_{j}^{{{\text{T}}_{{\text{k}}} }} } \right)} ,$$

where ${\text{D}}$ is a dissimilarity measure, $P_{i}^{{{\text{T}}_{{\text{k}}} }}$ ($P_{j}^{{{\text{T}}_{{\text{k}}} }}$) is the discrete probability distribution of call fractions for individual $i$($j$) at time period T_k_, with $i,j \in \left[ {1..n} \right]$, $i \ne j$, and ${\text{k}} \in \left\{ {1,2} \right\}$.

In the following, we denote by $x_{i}^{{{\text{T}}_{1} }} = \left( {x_{i,j}^{{{\text{T}}_{1} }} } \right)_{j = 1,n ; i \ne j}$ a sample of observations of size ${\text{n}} - 1$ corresponding to the *inter-individual* dissimilarities calculated between a given individual $i$ and each of the other individuals $j$ from population $A$, such as $i \ne j$, within period T_1_.

Similarly, we denote by $x_{i}^{{{\text{T}}_{2} }} = \left( {x_{i,j}^{{{\text{T}}_{2} }} } \right)_{j = 1,n ; i \ne j}$ a sample of observation of size ${\text{n}} - 1$ corresponding to the *inter-individual* dissimilarities calculated between a given individual $i$ and each of the other individuals $j$ from the population $A$, and such as $i \ne j$, within period T_2_.

**Step 5:** *Persistence assessment*. Persistence is assessed by comparing how the *intra-individual* dissimilarity of a given individual’s daily rhythm between T_1_ and T_2_ lies in comparison with the values of his *inter-individual* dissimilarities in each of the two time periods T_1_ and T_2_. A daily rhythm of telephone call activity is found persistent if and only if the *intra-individual* dissimilarity tends to minimize the set of *inter-individual* dissimilarities. For an individual $i$, the assessment consists of a sign test of quantile as follows:

**Step 5.1** First, We set $z_{i} = \left( {z_{i,j}^{{{\text{T}}_{{\text{k}}} }} } \right)_{j = 1,n ; i \ne j ;k = 1,2} = (1_{{\{ y_{{\text{i}}} {-}x_{i,j}^{{{\text{T}}_{{\text{k}}} }} { } > 0\} }} )_{j = 1,n ; i \ne j; k = 1,2}$, the vector resulting of the comparison of each *inter-individuals* dissimilarity $x_{i}^{{{\text{T}}_{{\text{k}}} }}$ in each period T_k_ with the individual $i$’s *intra-dissimilarity* $y_{i}$, where the dimension of the vector $x_{i}^{{{\text{T}}_{{\text{k}}} }}$ is n-1 and ${\text{k}} = \left\{ {1,2} \right\}$, which corresponds to $2n - 2$ comparisons. Each component of $z_{i}$ has values in {0, + 1}, where + 1 corresponds to a success for obtaining an inter-individual dissimilarity lower than an intra-individual dissimilarity, and 0 to a failure.

**Step 5.2** Let set ${\text{N}}_{i}^{ + } = {\Sigma }_{j = 1,n ; i \ne j, k = 1,2 } z_{i}^{{{\text{T}}_{{\text{k}}} }}$ being the total number of successes of individual $i$ in the two periods T_1_ and T_2_.

**Step 5.3** We set the null hypothesis H_0_ = {the probability for obtaining an *inter-individual* dissimilarity lower than an *intra-individual* dissimilarity is equal to $q$}, with $q$ set at ½ by default (case of median test).

Hence, under H_0_, each of the observed *inter-individual* dissimilarities has a probability $q$ for being lower than the *intra-individual* dissimilarity $y_{i}$. We thus have ${\text{N}}_{{\text{i}}}^{ + } { }\sim B\left( {2n - 2,q} \right)$, if the behavior of individuals *j* in period T_1_ and T_2_ can be considered as independent.

In this context, considering population A, and time periods T_1_ and T_2_, a significant P-value obtained from a binomial test brings a statistical element of response that supports the persistence of the daily rhythm of telephone call activity of individual $i$.

#### Persistence analysis for more than two successive time periods (N_T_ > 2)

Let a given population of $n$ individuals $A = \left\{ {i, i \in \left[ {1 \ldots n} \right]} \right\}$ observed over N_T_ successive periods of time. In this context, for a given individual $i \in A$, we evaluate his trend for having a persistent behavior at telephone between successive time periods.

To this end, we run the same persistence analysis than that described above for each two successive periods of time. We consider two types of events from the obtained results: (1) “Persistence” that corresponds to a significant p-value, and (2) “No Persistence” that corresponds to a non-significant *P-value*.

We consider the sample of observations $v$ of length N_T_ − 1, where each element of $v$ is at value in $\left\{ {0,1} \right\}$ with 1 (resp. 0) corresponding to a “Persistence” event (resp. “No persistence”) obtained from the comparisons. Finally, a sign test is applied to $v$. A significant result indicates individual $i$ tends to have a persistent behavior at telephone with regards to population $A$ and the N_T_ successive periods of time.

In practice, we used *P*-value < 0.05 in statistical tests as the level of significance. All statistical calculations were done in the R software environment (version 3.1.6; R Foundation for Statistical Computing, Vienna, Austria).

### Approval of the experimental protocol

The present study and its corresponding experimental protocols were approved by the French Data Protection Authority (CNIL registered data protection officer, France Telecom 2011 n°44). All experimental methods were carried out as per the relevant regulations and written informed consent was obtained from all participants before data were collected and anonymized.

## Results

We applied the analytical procedure proposed in the Methods section on a 12-month CDRs dataset of adults over 65 years. Three cases are tested separately: (1) outgoing, (2) incoming, and (3) total call activities. We considered a two successive temporal window (N_T_ = 2), and we used the Jenson-Shannon Divergence dissimilarity (denoted D in Methods section) as dissimilarity measurement.

**Figure 2:**
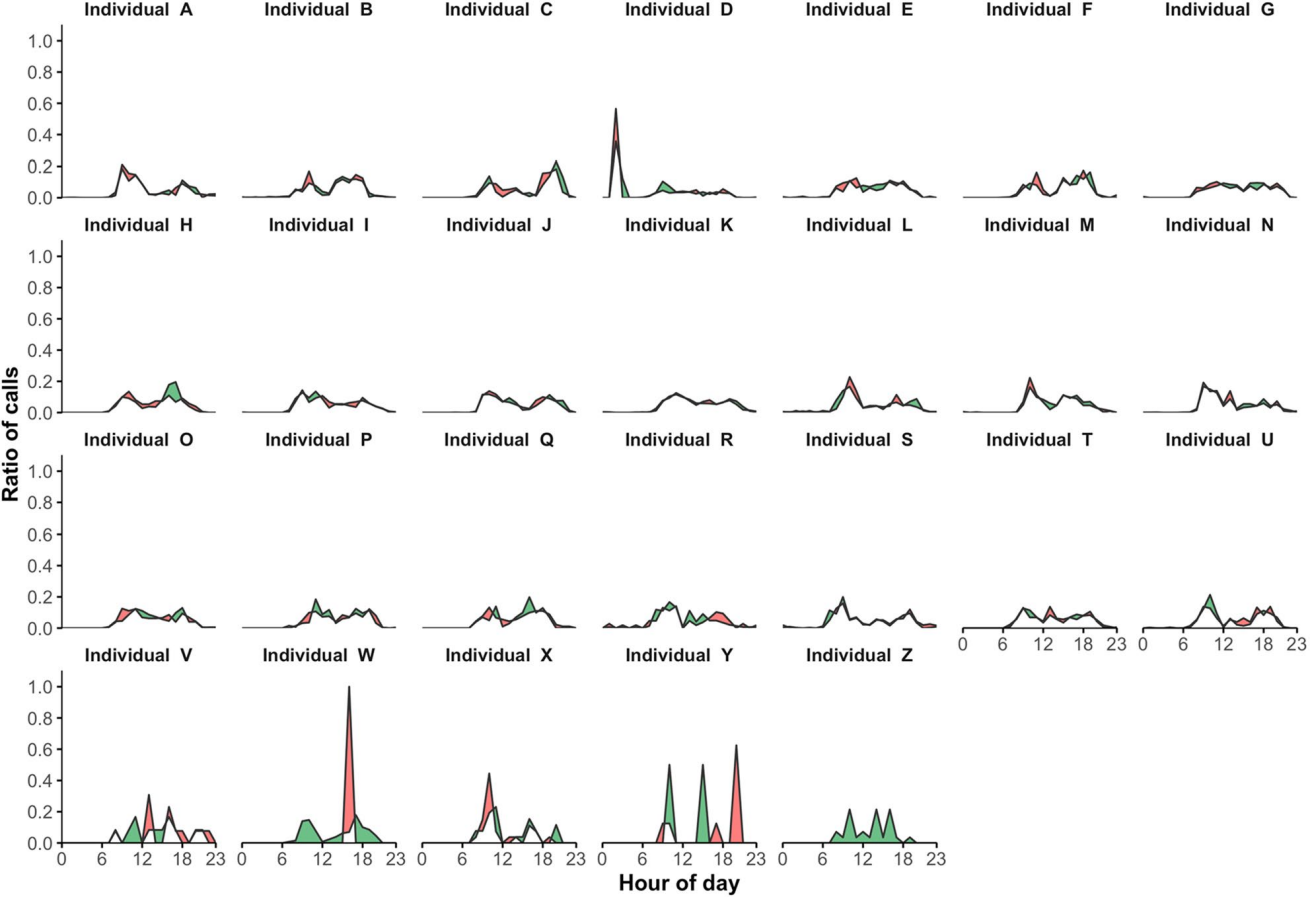
Comparison of two daily rhythms of outgoing telephone call activity. Daily rhythms of outgoing telephone call activity are calculated for the two successive time periods of 6 months each, T_1_ and T_2_. Their differences are illustrated by coloring the area between the two corresponding curves: (1) in green when the ratio of calls corresponding to T_1_ is higher than T_2_, and (2) in red for the opposite.

Figure 2 presents for each individual the two daily rhythms of outgoing telephone call activity for the two successive time periods of 6 months each, T_1_ and T_2_. The differences between T_1_ and T_2_ are illustrated by a colored area differentiating the two corresponding curves: (1) in green when the ratio of calls corresponding to T_1_ is higher than T_2_, and (2) in red for the opposite. Figure 2 illustrates the similarities and differences observed between two successive rhythms of telephone call activity of the same individual. On this figure, we can observe that some of them, such as individuals A or B for instance, seem to exhibit a few differences between T_1_ and T_2_. Others, such as individuals W or Y for instance, seem to exhibit more pronounced differences.

The statistical results are stored in Table 1. For a given individual *i*, N_i_^+^ informs on the number of his inter-dissimilarities lower than his intra-dissimilarity, whereas the corresponding p-value permit to associate N_i_^+^ with a *persistent* or *not persistent* nature that is statistically valid. For the present dataset, the statistical results stored in Table 1 show that individuals V, W, Y, Z do not present a circadian rhythm of telephone call activity that is significantly persistent (p-values > 0.05) against the population observed. On the contrary, the other individuals show a significant persistence (P-values < 0.05).

**Table 1:** Results of statistical tests of persistence.

Individual	Outgoing calls			Incoming calls			Total calls		
	P-value	N_+_	N_comp_	P-value	N_+_	N_comp_	P-value	N_+_	N_comp_
A	8.88E−16	0	50	8.88E−16	0	50	8.88E−16	0	50
B	8.88E−16	0	50	8.88E−16	0	50	8.88E−16	0	50
C	4.53E−14	1	50	8.88E−16	0	50	8.88E−16	0	50
D	8.88E−16	0	50	8.88E−16	0	50	8.88E−16	0	50
E	2.23E−10	4	50	2.81E−06	9	50	2.81E−06	9	50
F	8.88E−16	0	50	4.53E−14	1	50	8.88E−16	0	50
G	8.88E−16	0	50	8.88E−16	0	50	8.88E−16	0	50
H	5.82E−07	8	50	8.88E−16	0	50	2.23E−10	4	50
I	8.88E−16	0	50	8.88E−16	0	50	8.88E−16	0	50
J	8.88E−16	0	50	8.88E−16	0	50	8.88E−16	0	50
K	8.88E−16	0	50	1.30E−03	14	50	8.88E−16	0	50
L	4.53E−14	1	50	8.88E−16	0	50	8.88E−16	0	50
M	8.88E−16	0	50	8.88E−16	0	50	8.88E−16	0	50
N	8.88E−16	0	50	8.88E−16	0	50	8.88E−16	0	50
O	5.82E−07	8	50	8.88E−16	0	50	4.53E−14	1	50
P	1.13E−12	2	50	3.25E−02	18	50	4.51E−05	11	50
Q	1.30E−03	14	50	4.53E−14	1	50	8.88E−16	0	50
R	1.85E−11	3	50	1.13E−12	2	50	2.10E−09	5	50
S	8.88E−16	0	50	1.13E−12	2	50	8.88E−16	0	50
T	8.88E−16	0	50	2.81E−06	9	50	2.23E−10	4	50
U	8.88E−16	0	50	8.88E−16	0	50	8.88E−16	0	50
V	1.61E−01	21	50	8.88E−16	0	50	8.88E−16	0	50
W	1.00E+00	38	50	1.00E+00	48	50	1.00E+00	47	50
X	2.23E−10	4	50	4.53E−14	1	50	1.13E−12	2	50
Y	1.00E+00	48	50	8.88E−16	0	50	8.88E−16	0	50
Z	4.44E−01	24	50	9.68E−01	31	50	8.99E−01	29	50

The results comprise three different cases: (1) outgoing, (2) incoming, and (2) total calls test of persistence. The p-value corresponds to the one obtained with a sign test. N + correspond to the number of inter-individual dissimilarities lower than the intra-dissimilarity. N_comp_ corresponds to the number of comparisons assessed.

**Figure 3:**
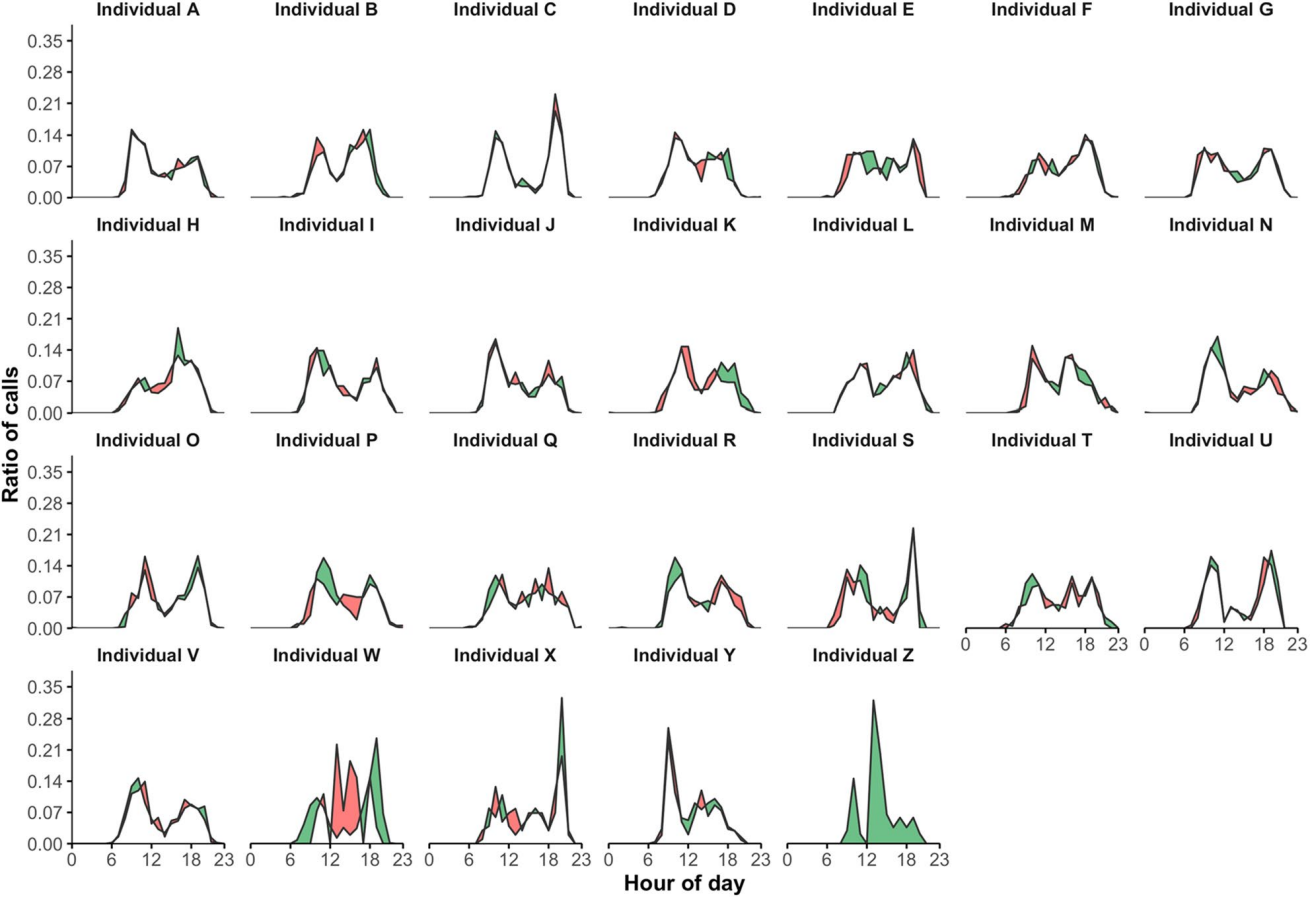
Comparison of two daily rhythms of incoming telephone call activity. Daily rhythms of incoming telephone call activity are calculated for the two successive time periods of 6 months each, T_1_ and T_2_. Their differences are illustrated by coloring the area between the two corresponding curves: (1) in green when the ratio of calls corresponding to T_1_ is higher than T_2_, and (2) in red for the opposite.

Figure 3 presents for each individual the two daily rhythms of his incoming telephone calls for the two successive time periods of 6 months each, T_1_ and T_2_. Again, the statistical results are stored in Table 1. It appears that W and Z are the only ones who do not show significant persistence (P-values < 0.05). For individuals V and Y, this implies that the persistent nature of their circadian rhythms of telephone call activity depends on the nature of the direction of calls considered.

**Figure 4:**
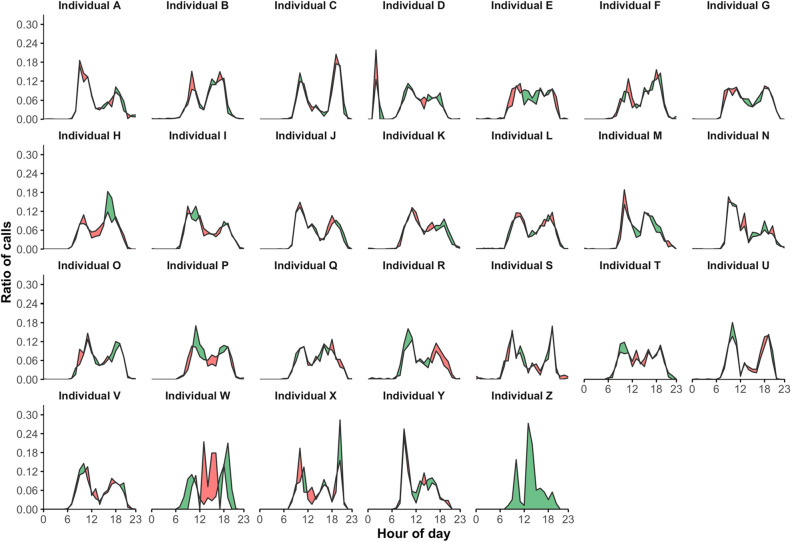
Comparison of two daily rhythms of total telephone call activity. Daily rhythms of total telephone call activity are calculated for the two successive time periods of 6 months each, T_1_ and T_2_. Their differences are illustrated by coloring the area between the two corresponding curves: (1) in green when the ratio of calls corresponding to T_1_ is higher than T_2_, and (2) in red for the opposite.

Figure 4 presents for each individual the two daily rhythms of his total telephone call activity for the two successive time periods of 6 months each, T_1_ and T_2_. The statistical results are stored in Table 1. Again, W and Z are the only ones who do not show significant persistence (P-values < 0.05).

## Discussion

The present study has been carried out for addressing the absence of statistical consistency in the current methods of persistence analysis applied to the circadian rhythms of telephone activity. To this end, we show that the methods currently used in the literature can be reformulated into a well-defined and simple non-parametrical statistical problem. In this train of thought, we propose a novel statistical approach that permits to measure the circadian rhythms of telephone activity using a sign test of quantiles and which permits to ensure the results’ statistical validity. Then, we illustrate an application of this statistical procedure by assessing the persistence of the circadian rhythms of telephone call activity on a 12-month CDRs dataset of adults over 65 years. Three cases are tested separately: (1) outgoing, (2) incoming, and (3) total call activities. On the whole, the results show the ability of our approach for assessing persistence with a statistical significance. In particular, it permits to figure out that, in this CDRs dataset, even if most of the older individuals from the observed population show a significant persistent circadian behavior at telephone, this observation (1) does not stand systematically and statistically for every older individuals, and (2) may depend on the nature of the direction of calls considered in the analysis (outgoing, incoming or total calls).

Thus, as such, the statistical method we propose in this paper can be beneficial for all studies involving the persistence assessment for telephone call activity. In fact, the current methods used for assessing persistence in literature are mainly inherited from both (1) the field of complex network sciences^38^—with a physical approach known as social physics^15^—and (2) the field of computational social sciences^39,40^. Such methods hence rely rather on (1) the use of simple mathematical estimators integrated within a sophisticated formalistic scientific approach proper to social physics, and (2) a careful work of observation and interpretation of results which is proper to the field of social sciences than on a proper statistically consistent approach^41^. For applications associated with the understanding human social behavior, the combination of approaches (1) and (2) permits to address complex scientific questions on an original and relevant way. In particular, in the studies related to the telephone activity, mixing together quantitative and more qualitative approaches permits to bring relevant results relying on both objective elements of observation and on a careful work of interpretation, as evinced in^21,25,30^ for instance. Following this train of thought, integrating such an approach into a broader analytical process which includes statistical validation permits to introduce a certain level of significance regarding both the objective and subjective elements proposed and discussed in the involved studies. In particular, such a statistical assessment permits to nuance the results’ interpretations proposed by the researchers by producing statistically valid elements that reinforce, or invalidate, the investigated theories. Such an inductive reasoning approach, which can seem evident for researchers working in the field of statistics, may be of a strong importance for new hybrids fields of computational science, such as social physics and computational social sciences.

Furthermore, it is interesting to mention that the novel statistical approach we propose in this paper can be situated into a broader, legitimate, scientific approach. In particular, regarding the combination of statistics with the concept of *inter-intra* dissimilarities used in the present study and introduced by Saramäki et al.^21^, such an approach has a long history and still remains of a strong importance in the fields of statistics and data analysis. Already at the beginning of the XX^th^ century, biometricians proposed to consider the *inter-intra* approach for comparing the mean effects observed in samples of interest. For instance, this was the case of Fisher who introduced the total variance decomposition that permits to distinguish the *intra* and *inter* group variances^42^. This approach is at the origin of the analysis of variance (ANOVA), which is, and remains to be, an essential approach for various domains of research requiring statistical evidences, such as the biomedical research sector for instance. Interestingly, this concept of *inter-intra* is also present at the core of recent data analysis approaches. For instance, in the field of statistical learning, unsupervised machine-learning methods usually rely on this concept for measuring the consistence of clusters obtained after the statistical analysis^43^. Thus, as such, the novel statistical approach we propose in this paper can benefit the complex and emerging approaches of persistence analysis used in the current literature by fitting this last one into a broader and coherent scientific framework.

Then, beyond these contextual elements underlying the framework of our approach, at least two elements of interest can be advanced for justifying the specific use of our statistical procedure in *mHealth* studies. First, it can inform with a certain level of significance about the personal nature of an individual’ social behavior observed at telephone. For circadian rhythms specifically, there are yet evidences of singularity for the circadian periods of an individual^1^. Despite the precise and maintained entrainment of the circadian rhythms of activity on a 24-h clock, such an individual characteristic plays an essential role in the individual’s life, and it is of strong importance in the field of chronobiology—and more broadly in personalized medicine—for addressing a patient’s need adequately in time^7^. At the social level, the statistical procedure we propose can permit to address this essential point from a social and statistical view. Second, the *objective* and *personal* nature of data provided by modern phone technologies is presupposed in various fields of application related to personalized health^13,36^. Often, this assumption is used as an argument of interest regarding the use of such technologies in health (see recent reviews on the subject for instance^13,36,44^). As such, the statistical procedure we propose can permit to assess such a pre-notion of digital signature with regards to the daily social interactions occurring at telephone by assessing both their robustness and distinctiveness in time. For the clinical practice, such an assessment is of a strong importance before validating the personal nature of a patient’s data generated by his phone device and, *a fortiori,* the personalized nature of the digital solution related to.

At this stage, however, it is important to recall that persistent analysis does not represent the only way to model consistent or inconsistent circadian rhythms from activity data. For instance, in another study^45^, Luque-Fernandez et al*.* showed how an absence of circadian rhythms of a given physical activity can be modeled, by using alternative approaches than persistent analysis. In particular, these authors showed, in a totally different context, how specific behaviors such as labor can be modeled by fitting data about the number of labors at a given hour to a log-link trigonometric Poisson model. In particular, they investigated how such model can be used for detecting a damped sinusoidal behavior and how an observational Fourier analysis can permit to identify the presence of a stable periodicity. This approach was well adapted to the data observed in this study^45^ presenting an important variance. Here, the weak value of the variation coefficients (standard deviation/mean) of the data (equal for example to about 1/4 for the reference- and self-distances used for comparing the daily phone activities) authorizes to use the average daily curves of phone activity as representative of a persistent highly not sinusoidal rhythm without estimating its Fourier fundamental and harmonic components. Taken together, these elements can underline the complementarity of persistence analysis with alternative ones, depending on the data analyzed and the nature of the activity to which they refer.

Thus, as such, the statistical procedure proposed in this study can be used for completing current methods of persistence analysis and for assessing the statistical validity of the existing results reported in the literature related to CDRs analysis. More importantly, it provides an opportunity to open up CDRs analysis to various domains of application that require an access to statistical significance, including health care monitoring. Finally, for health professionals, social information provided by the continuous, objective, unobtrusive analysis of persistent circadian rhythms of telephone activity can be used in complement of traditional punctual, subjective clinical questionnaires which require the active participation of the patient^46,47^. In the field of health care monitoring, such a use of phone technologies can permit to enhance the general framework around the analysis of the circadian rhythms of activity of an individual through time, which are of various natures. In particular, this enhancement is induced by the fact that there is no pure independency between the biological, physiological and social manifestations of the circadian rhythms^4^. On the contrary, these last ones are deeply entangled with each other. This complex interplay results in profound dependencies connecting the different levels of human life^4^. In particular, as reported by Social Zeitgeber theory^48^, a biological alteration of the circadian rhythms can have adverse repercussions on social rhythms, and inversely. More importantly, such transverse alterations may act as a catalytic retroactive process that worsens the initial disruption which occurred in the individual’s life. In this train of thought, there are evidences of significant associations between the occurrences of circadian rhythms’ biological or social disruptions and various adverse situations including, but not limited to, mood disorders^48^, social jet-lag^49^, sleep disturbances^50^ or cognitive dysfunctions^51^, just for naming a few. Hence, in the field of health care monitoring, a deep comprehension of social manifestations of circadian rhythms can be of strong importance to properly analyze the circadian rhythms considering all their complexity and, then, for better managing a patient’s health over time to prevent, when it possible, the occurrence or worsen of specific symptoms or diseases related to circadian rhythms alterations.

From the present work, a relevant perspective could consist of using the statistical analysis of persistence we propose for longitudinal studies in order to measure the robustness and distinctiveness of the circadian rhythms of telephone call activity of a population suffering from a specific illness or chronic disease such as bipolar disorder. Such an approach could permit to quantify the individuals’ social rhythm regularity regarding specific disruptions. Such work could contribute to better analyze the role and manifestation of circadian rhythms of a social nature in health and to better understand their entanglement with biological and physical cues involved in the circadian rhythms of activity.
